# Reduplicated medial parapatellar plica: a case of a medial plica anatomical variation recalcitrant to conservative treatment

**DOI:** 10.1007/s00276-022-03025-3

**Published:** 2022-09-30

**Authors:** Theodorakys Marín Fermín, Luca Macchiarola, George Tsakotos, Ioannis Terzidis, Trifon Totlis

**Affiliations:** 1 Thessaloniki Minimally Invasive (The-MIS) Orthopaedic Center, St. Luke’s Hospital, Thessaloniki, Greece; 2 Aspetar Orthopaedic and Sports Medicine Hospital, Doha, Qatar; 3 Departimento di Medicina Clinica e Sperimentale, Università degli Studi di Foggia, Foggia, Italy; 4 Department of Anatomy, Faculty of Health Sciences, School of Medicine, National and Kapodistrian University of Athens, Athens, Greece; 5 Department of Anatomy and Surgical Anatomy, Faculty of Health Sciences, School of Medicine, Aristotle University of Thessaloniki, P.O. Box 300, 54124 Thessaloniki, Greece

**Keywords:** Plica syndrome, Medial parapatellar plica, Cartilage injury, Synovium, Knee anatomy

## Abstract

**Purpose:**

The current study aims to report the radiologic and clinical appearance of a rare anatomical variation of the knee medial synovial plica along with its response to conservative and surgical treatment.

**Case presentation:**

This report portrays a 29-year-old male patient with anteromedial gradual onset right knee pain, aggravated when descending stairs or prolonged sitting. Physical examination revealed medial parapatellar local tenderness, a palpable click in this area when the knee was extended, and hamstring tightness. Magnetic resonance imaging showed a duplicated medial plica, characterized by a high-intensity signal of the infrapatellar fat pad medial portion, after which a presumptive diagnosis of medial plica syndrome was proposed. After conservative treatment failure, the patient underwent standard knee arthroscopy that revealed a superior low profile and an inferior high profile medial plica, and hypertrophy of the medial portion of the infrapatellar fat pad. Both plicae and fat pad were resected with a mechanical shaver until no contact between the femoral trochlea and the fat pad was observed during full range of motion. At 4 weeks postoperatively, symptoms completely resolved, and the patient was allowed to return to full activity with no recurrences at 1 year follow-up.

**Conclusions:**

The current study presented a rare anatomical variation of the knee medial synovial plica that was symptomatic and recalcitrant to conservative treatment. This case report may be useful for radiologists and orthopaedic surgeons to differentiate this special plica type and consider its response to conservative and surgical treatment during patient management.

## Introduction

The medial parapatellar plica is a thin and elastic synovial fold with a membranous pearl-white appearance and varying thickness and is present in up to 50% of the individuals [23]. It runs obliquely from the medial knee capsule to the synovium of the infrapatellar fat pad [3, 23]. Mayeda described it for the first time in 1918 and has been named with a vast terminology: Iino’s band, chorda cavi articularis genu, shelf, plica alaris elongata, plica synovialis mediopatellaris, among others [5, 21, 23].

The presence of plicae can lead to repetitive trauma, and inflammation compromising the cartilage of the medial femoral condyle, producing medial and/or anterior knee pain, snapping, and effusion, and referred to as medial parapatellar plica syndrome, mimicking meniscus tear or patellofemoral syndrome-like symptoms [10, 14, 25].

The current study aims to report the radiologic and clinical appearance of a rare anatomical variation of the knee medial synovial plica along with its response to conservative and surgical treatment. The present case report was presented following the CARE guideline [22].

## Case presentation

This is the case of a 29-year-old male chef that complained about anteromedial gradual onset pain of the right knee for the 2 months, aggravated when descending stairs and when bending the knee over 90° of flexion for more than a minute. The patient did not report any relevant medical history, family background, or previous surgical intervention in the knee.

On physical examination, the patient presented medial parapatellar local tenderness and palpable click in this area when the knee was extended. The hamstrings 90/90 straight leg raise test revealed a 25° of extension deficit in the right lower limb.

**Fig. 1:**
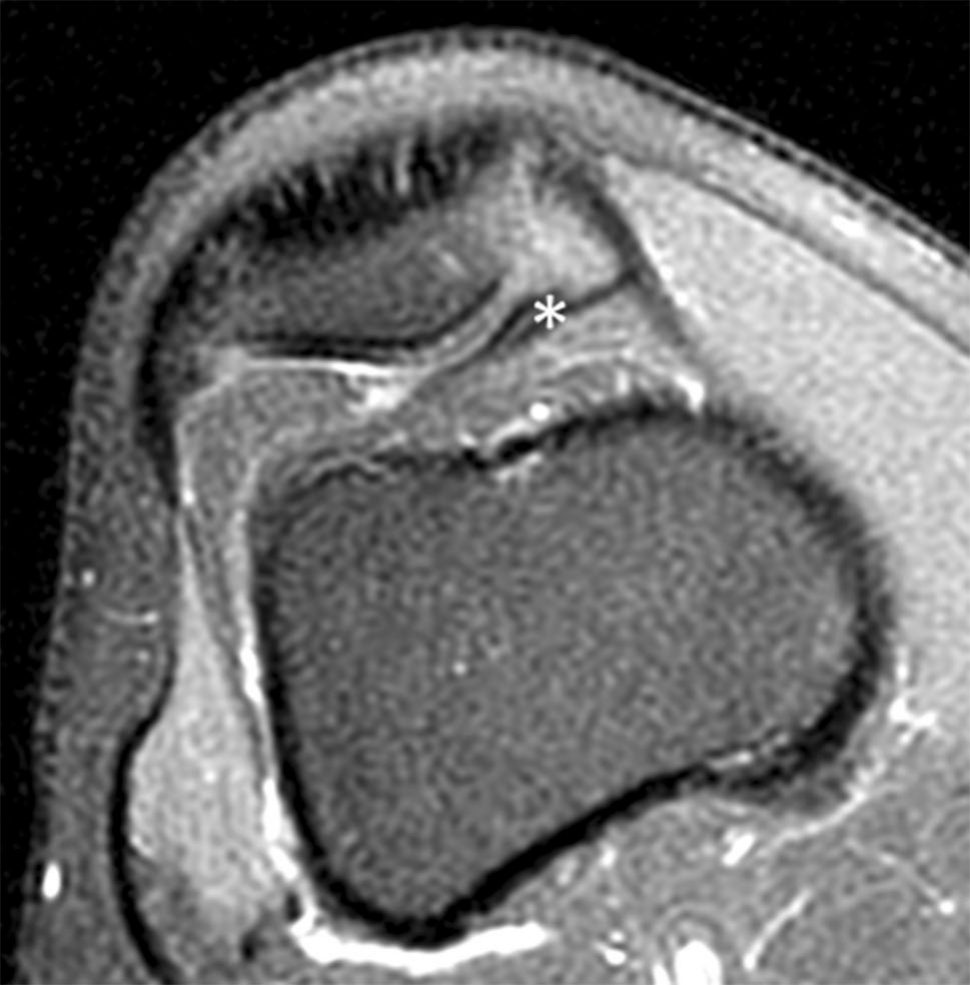
Magnetic resonance imaging of a duplicated medial plica (asterisk)

Diagnostic imaging revealed a normal right knee X-ray. The patient underwent magnetic resonance imaging (MRI) at 1.5 Tesla (General Electric Signa HDxt). He was positioned supine with the knee in a 10° flexion and 15° external rotation. The examination protocol included axial, coronal, and sagittal PD-WI, sagittal T2–WI, coronal T1–WI, and STIR MR sequences, all with a slice thickness of 4 mm. No intravenous media contrast was administered. Imaging revealed a duplicated medial plica (Fig. 1), characterized by a high-intensity signal of the infrapatellar fat pad medial portion, after which a presumptive diagnosis of medial plica syndrome was proposed.

**Fig. 2:**
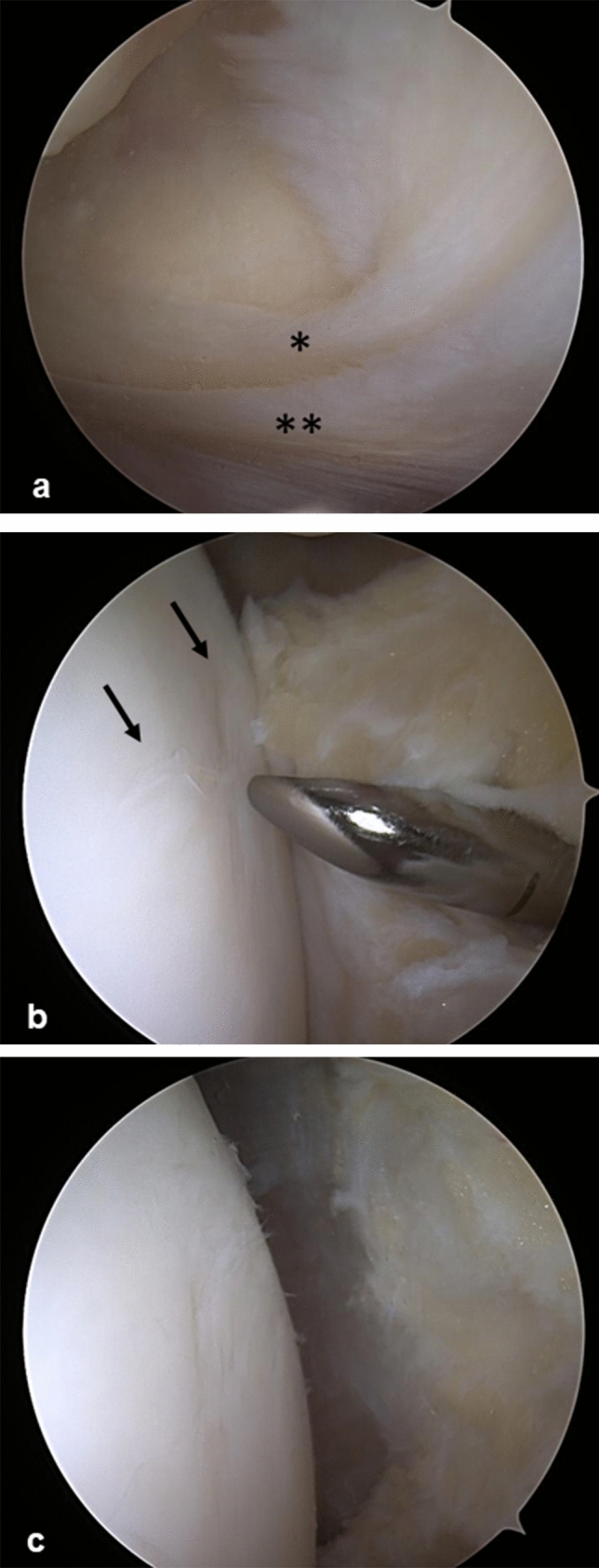
Arthroscopic management of the case. **A** Superior low profile (single asterisk) and an inferior high profile (double asterisks) medial plica. **B** Plicae and the hypertrophied fat pad were resected using a shaver. The arrows show the superficial “sand-mark” fissures on the articular cartilage. **C** Arthroscopic appearance after plicae and fat pad resection

The patient was suggested to undergo a 12 week conservative treatment trial including NSAIDs and a rehabilitation program including analgesic physical therapy, hamstrings stretching and manual therapy, followed by quadriceps, gluteus muscles, and hip external rotator muscles strengthening and core stability. Despite the promising initial outcomes, the symptoms did not disappear completely, especially during stair descending and prolonged sitting. Therefore, surgical treatment was scheduled. The patient underwent standard knee arthroscopy (Arthrex, Naples, FL, USA) under general anesthesia, which revealed a duplicated medial plica, including a superior low profile and an inferior high profile medial plica (Fig. 2A), and hypertrophy of the medial portion of the infrapatellar fat pad (Fig. 2B). During knee flexion beyond 40°, the high-profile medial plica along with the hypertrophied fat pad came in contact with the medial femoral condyle proximal portion, which presented with superficial fissures at the articular cartilage (Fig. 2C). No other intra-articular pathology was found. Both medial plicae and the fat pad hypertrophy were resected with a mechanical shaver until no contact between the femoral trochlea and the fat pad was observed during the range of motion (Fig. 2D). The patient was discharged on the same day and allowed full weight-bearing as tolerated on the affected limb. Subsequently, he followed a postoperative rehabilitation program to regain full range of motion, stretching hamstring muscles and strengthening quadriceps and gluteus muscles. At 4 weeks postoperatively, symptoms completely resolved, and the patient was allowed to return to full activity. After 1 year of postoperative follow-up, the patient is still asymptomatic.

## Discussion

Medial parapatellar plicae are found in up to 79.9% of patients undergoing knee arthroscopy but are asymptomatic in most cases [17]. Sakakibara proposed a four-type medial parapatellar plicae classification [23]. Type A, cord-like (8.89%); type B, shelf-like but without medial femoral condyle coverage (35.56%), type C, large shelf-like, which covers the anterior medial femoral condyle (51.11%); and type D, characterized by a double insertion (4.44%) [23].

Although a medial parapatellar plica is the norm in the knee anatomy, its most infrequent type D can be considered an anatomical variation, even more, when it accounts for 10% of the symptomatic cases [8, 10, 17, 20, 23, 27]. Type C and D plicae, are more likely to be symptomatic, and they have been correlated with anteromedial patellofemoral cartilage damage in up to 82.9% of the patients, ranging from ICRS I to IV injuries [2, 7, 8, 10, 21]. The present case report portrays a symptomatic medial parapatellar plica type D with a characteristic medial femoral condyle cartilage “sanding-mark”-like ICRS grade I injury [8], a surgically confirmed cause of anterior knee pain.

The embryological origin of the knee plicae is still controversial [11, 12, 23]. However, the most accepted theory establishes the development of menisci, cruciate ligaments, and a partitioned joint cavity in the 8 week fetus, with the subsequent fusion of the joint knee compartments and resorption of the synovial septum by the end 12th week. In cases of incomplete resorption of the synovial septum, residual mesenchymal tissues result in the knee plicae [5, 6,11, 12, 13, 19, 26].

The reduplicated medial parapatellar plica is a pair of oblique shelf-like synovial pleats. They extend from the genu articularis muscle to the knee joint medial wall to the infrapatellar pad with a double insertion. The plica presents two borders, medial and lateral; two extremities, superior and inferior; and two surfaces, anterior and posterior. The medial margin originates from the medial patellar recess, and the lateral or free border is concave towards the center of the joint. The superior extremity arises from the suprapatellar plica or neighboring synovial tissues, while its inferior extremity expands to the infrapatellar fat pad. Thus, the plica lies between the medial patellar articular surface and the medial femoral condyle [5, 8, 9, 17, 23, 25]. Histologically, it shows fibrous and chronic inflammation characteristics, most probably due to repetitive trauma during knee motion [2].

In addition, its thick and stiff consistency impinges through its free edges on the anterior facet of the medial femoral condyle and medial patella [17]. The plicae engage with the medial femoral condyle between 30 and 60° of knee flexion, starting with the inner flap of type D plica and even locking into the patellofemoral joint [10, 14]. This repetitive motion results in tenderness, swelling, snapping, locking sensation, potential chondral injuries, and further degeneration in the patellofemoral joint [5, 10, 12, 13].

Magnetic resonance imaging helps guide the diagnosis, showing sensitivity and specificity as high as 93% and 81%, respectively [10, 16, 24]. Our findings reflect the characteristic magnetic resonance findings, including the presence of a low-intensity cord or shelf on T2-weighted sagittal and axial images, especially during knee effusion [8]. Conservative treatment is the initial approach, including rest, pain and anti-inflammatory medication, physiotherapy, and corticosteroid injections, showing satisfactory outcomes, especially for younger patients with acute symptomatology and without cartilage involvement [1, 4, 15, 18].

The presence of a type C and D medial parapatellar plicae has been associated with conservative treatment unresponsiveness, longer duration of symptoms, and associated patellofemoral cartilage injuries, such as the patient presented in the current case report [10]. A meta-analysis by Gerrard et al. [5] reported favorable patient outcomes in medial parapatellar plica syndrome patients undergoing arthroscopic excision after failed conservative treatment. In the present study, arthroscopic media plica excision led to complete symptoms resolution at 1 month postoperatively, and the patient was then allowed to return to full activity, with no recurrence at the last follow-up, 1 year postoperatively.

A certain limitation of the study is the absence of pre and post operative patient reported outcome scores to objectively document the improvement. However, the patient post-operatively reported complete symptoms resolution and full return to previous activities. Moreover, primary aim of the study was not the clinical outcomes but to document the clinical relevance of this intra-articular anatomical variation of the knee.

This case report is helpful for radiologists and orthopaedic surgeons to recognize this special plica type on MRI and discriminate from other types. Differential diagnosis of duplicated plica from other types is important for daily clinical orthopaedic practice during decision-making with patients. As this case showed, the duplicated plica is recalcitrant to conservative treatment, whereas arthroscopic resection provides definite treatment followed by a short rehabilitation timeline to resume pre-operative activities.

## Conclusions

The current study presented a rare anatomical variation of the knee medial synovial plica that was symptomatic and recalcitrant to conservative treatment. MRI can reveal the reduplicated medial plica and arthroscopy provides the definite treatment. This case report may be useful for radiologists and orthopaedic surgeons to differentiate this special plica type and consider its response to conservative and surgical treatment during patient management.

## Data Availability

Not applicable.
